# Size and isolation of naturally isolated habitats do not affect plant-bee interactions: A case study of ferruginous outcrops within the eastern Amazon forest

**DOI:** 10.1371/journal.pone.0238685

**Published:** 2020-09-11

**Authors:** Carlos Eduardo Pinto, Marcelo Awade, Mauricio Takashi Coutinho Watanabe, Rafael M. Brito, Wilian F. Costa, Ulysses M. Maia, Vera L. Imperatriz-Fonseca, Tereza Cristina Giannini

**Affiliations:** 1 Instituto Tecnológico Vale, Pará, Brazil; 2 Instituto de Biociências, Universidade de São Paulo, São Paulo, SP, Brazil; 3 Universidade Federal do Pará, Pará, Brazil; University of the Balearic Islands, SPAIN

## Abstract

Pollination may be severely affected by the decreasing size and increasing isolation of habitat patches. However, most studies that have considered the effects of these two variables on plant-pollinator interactions have been carried out in areas that have undergone anthropogenic fragmentation, and little is known about their effects in natural habitats. The Carajás National Forest and Campos Ferruginosos National Park are two protected areas in the eastern Amazon where one can find isolated ferruginous outcrops characterized by iron-rich soil and herbaceous-shrub vegetation surrounded by Amazon forest. These patches of canga provide an opportunity to analyze plant-pollinator interactions in naturally fragmented areas. Our objective was to test whether the size and isolation of naturally isolated outcrops located in Carajás affect plant-pollinator interactions by using pollination syndromes and interaction networks. We determined the pollination syndromes of 771 plant species that occurred in eleven canga patches and performed field work to analyze plant-pollinator networks in nine canga patches. The structure of the plant-pollinator networks was not affected by the size or isolation of the canga patches. Generalist species were present in all canga areas, indicating that they are important in maintaining the plant communities in isolated canga patches. The lack of significance related to the distance between canga patches suggests that the forest does not prevent pollinator movement between canga patches.

## Introduction

Habitat fragmentation is one of the major threats to biodiversity [1]. Fragmented landscapes with low connectivity and covered by degraded or poor native vegetation can face extinction cascades, especially if keystone species or entire functional groups of species are lost [2]. The negative effects of fragmentation on biodiversity have been discussed for several taxa, such as plants [3], birds [4], reptiles and amphibians [5], and insects [6], and at different trophic levels [7]. However, more recently, fragmentation has been the subject of controversy because other studies have indicated that fragmentation can be considered a landscape-scale phenomenon *per se* [8, but see 9].

Most of the studies that have explicitly considered fragmentation were carried out in human-modified regions [10, 11], and studies in naturally isolated habitats are scarce. For example, in naturally isolated temperate dry grasslands, no significant relationship was found between landscape spatial structure and total species richness, but specialist species richness was affected [12]. Another study conducted in seminatural temperate grasslands revealed differences in beetle assemblages depending on the adjacent habitat type; species richness was higher and evenness was lower in grasslands adjacent to crop fields compared with grasslands adjacent to coniferous forests [13]. To the best of our knowledge, no studies have been conducted on naturally isolated tropical habitats.

Mutualistic bee-plant interactions can be analyzed through interaction networks, where interactions between partner species are represented as links within complex webs [10]. Previous works showed the impact of fragmentation on interaction networks [14], with detrimental consequences on pollination [15]. Flower visitation rates and plant fecundity in small and isolated habitats are reduced [16], which can be explained by declines in the abundance and diversity of pollinators under such conditions. Given that the transfer of pollen to conspecific stigmas (pollination) is a pollinator-density-dependent process, pollen transfer is expected to decrease in small populations [17, 18]. In addition, habitat fragmentation affects species richness, abundance, phenology, and reproductive success and the degree (number of links) of species generalization/specialization [19, 20]. On the other hand, larger areas present a greater diversity of pollination systems [21]. Other studies have shown that bee pollination is affected by landscape structure at different spatial scales [22, 23] and that habitat fragmentation affects the phylogenetic structure [24] as well as the topology of plant-pollinator networks ‘[25]. In smaller and/or more isolated fragments, specialist species should not be frequent or should not exist, and the network should be less specialized and exhibit less nestedness than networks in larger areas [10].

Mutualistic bee-plant interactions can also be analyzed in terms of pollination syndromes, which are defined as sets of floral traits, such as flower shape, color, and odor; nectar quantity and pollen location [26, 27], and are useful for identifying the most important pollinator of a plant species [28]. Thus, a high diversity of pollination syndromes reflects a relatively high functional diversity of pollination modes. In fact, the functional diversity of pollination modes is key to ecosystem persistence [29] and can be defined as the variety of life-history traits presented by an assemblage of organisms [30]. Floral traits are an important factor structuring pollination networks [31] and are responsible for mediating possible interactions [32]. Additionally, only within the possible interactions (morphological fit) will abundance be important in explaining the interactions formed [32].

Fragmentation can have particularly important effects on plant reproductive systems [33, 34] and pollination syndromes [21]. For example, it was demonstrated that the frequency of pollination syndromes was affected by fragmentation [20, 21, 35] and that plants with an anemophilous syndrome were more likely to persist in fragmented areas than plants with an entomophilous syndrome [20, 35]. In addition, compared with larger habitats, smaller habitats were found to show a reduced frequency of more specialized pollination syndromes (such as those associated with bats, birds, butterflies, and hawkmoths) and an increase in the proportion of tree species pollinated by diverse small insects (entomophilous syndrome, i.e., generalist vectors) [21].

Despite the rapid fragmentation of the eastern Amazon [1], studies on plant-pollinator interactions in the region are lacking. To fill this gap, our study was conducted in two protected areas, the Carajás National Forest and Campos Ferruginosos National Park (hereafter Carajás), located in the eastern Amazon. Both are covered by tropical Amazon forest, but series of ferruginous outcrops can be found on the tops of plateaus and ridges. These outcrops are locally known as ‘cangas’ and are located 650–800 m above sea level. They form natural patches surrounded by forests, with different sizes and different levels of isolation, i.e., presenting varying distances from one another. Canga soil is shallow and rich in iron [36], presenting open vegetation predominately consisting of herbs and shrubs [37], in striking contrast with areas of the Amazon forest, which contain large trees reaching up to 60 m [38].

We tested whether size and isolation affect bee-plant interactions in naturally isolated ferruginous outcrops located in Carajás. More specifically, we asked two main questions:

1) Do the size and isolation of canga patches explain pollination syndrome frequency and plant composition? To answer this question, we verify whether the number of plants classified as showing a certain syndrome varies with the size of the canga patches and their isolation (i.e., the distance between them). We expect that the frequency of plants presenting pollination syndromes associated with birds, bats, butterflies and hawkmoths will decrease in smaller and more isolated fragments compared with larger and less isolated fragments. On the other hand, we expect that pollination by bees, diverse small insects and wind will be more frequent in small and more isolated fragments.

2) Do the size and isolation of canga patches affect interaction network structure and the diversity of interactions and pollinators? To answer this question, we verify whether nestedness, specialization, pollination diversity and interaction diversity vary with the size of canga patches and their isolation. We expect that pollination networks in smaller and more isolated fragments will have lower specialization, nestedness, pollination diversity and interaction diversity than those in larger and less isolated fragments.

## Methods

This project was carried out in the Carajás National Forest with permission from IBAMA (SISBIO 48272–3, 63324–1).

### Study area

**Fig 1:**
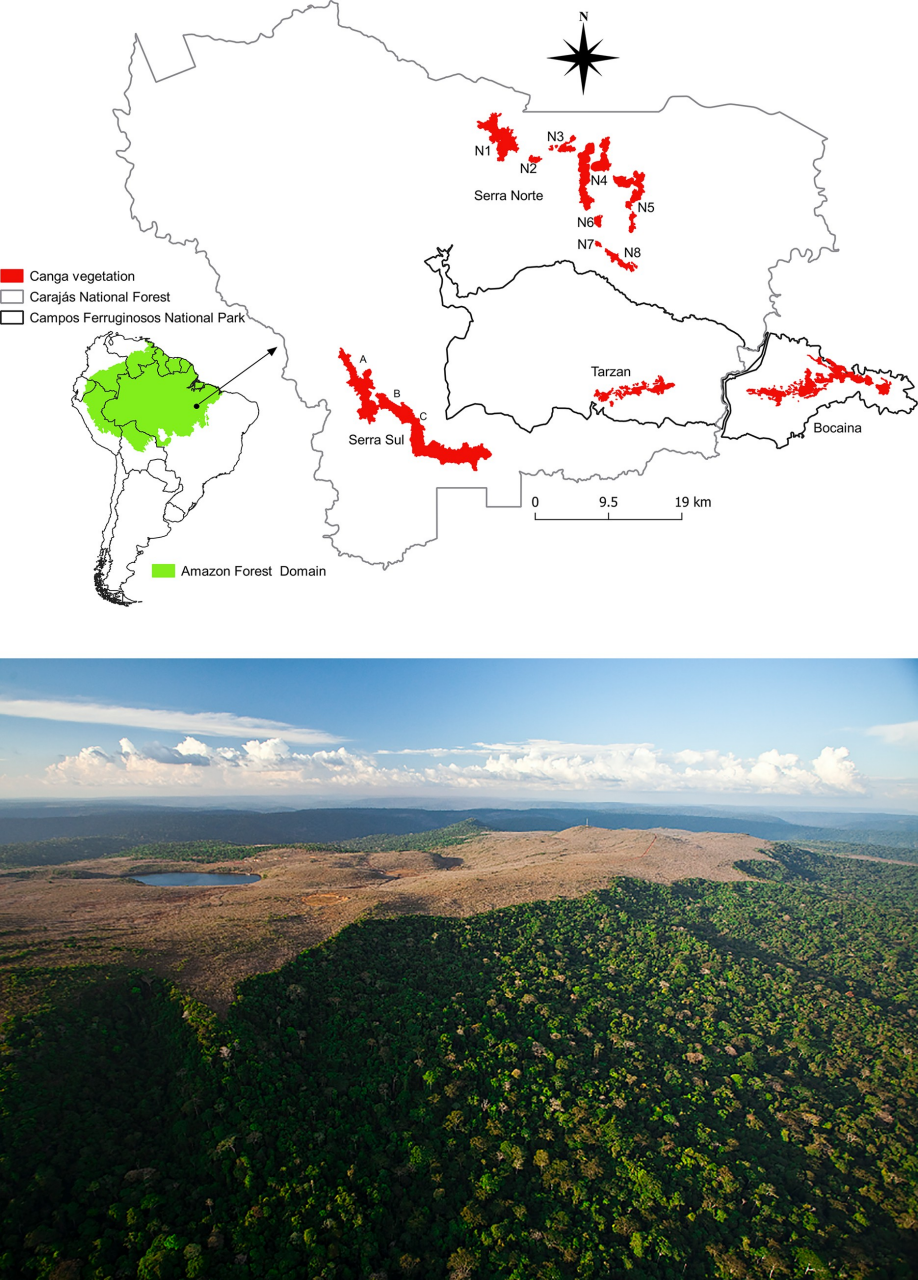
Above: Study area of the ferruginous outcrops located within the tropical Amazon forest of Carajás. Below: Naturally isolated ferruginous outcrops surrounded by the tropical Amazon forest (photo: João Marcos Rosa).

The National Forest of Carajás and National Park of Campos Ferruginosos (hereafter Carajás) are located in the southeastern state of Pará (05°52’S–06°33’S, 49°53’W–50°45’W; Fig 1). The patches of ferruginous outcrops (hereafter cangas) are located on some hilltops within the forest and present high solar incidence, high and constant temperatures throughout the year (average of 26°C) and seasonal variation in precipitation totaling more than 2000 mm per year [39]. We used QGIS software (Open Source Geospatial Foundation Project) to calculate the size of each canga patch (Table 1). To determine the isolation of canga patches, we used the nearest neighbor distance function in PostGIS software (Spatial and Geographic Objects for PostgreSQL).

**Table 1:** Plant species and pollination syndromes in each area of the ferruginous outcrops (cangas).

Area	Anemo-phily	Cantharo-phily	Entomo-phily	Phalaeno-phily	Melitto-phily	Ornitho-phily	Psycho-phily	Chiroptero-phily	Size (ha)	Isolation (m)	Number of plant species
Bocaina	55	3	26	7	109	11	4	5	2,145	25,570	220
n1	71	8	37	14	190	28	7	5	1,181	1,755	360
n2	20	1	11	6	66	9	5	0	86	1,490	118
n3	50	4	19	6	106	15	4	3	210	658	207
n4	61	5	28	2	143	16	5	3	1,484	658	263
n5	52	3	26	7	136	16	7	4	826	954	251
n6	26	1	11	2	45	5	4	1	99	1,302	95
n7	28	1	11	1	53	6	3	1	255	549	104
n8	25	1	11	1	49	7	1	1	33	549	96
Serra Sul	99	12	62	20	371	31	9	8	4,638	14,779	612
Tarzan	41	1	23	6	115	12	6	2	865	9,325	206

Carajás contains four main hills (Serra Norte, Serra Sul, Bocaina and Tarzan). We considered Serra Sul in its entirety for the first question and divided this area into three parts to address the second question (namely, S11A, S11B and S11C). We implemented this approach because the isolation of the parts of Serra Sul is not as evident as that for Serra Norte (Fig 1). Thus, we considered that the distance between the areas would not influence the distribution of floral traits and consequently pollination syndromes. On the other hand, in terms of the observations of floral visitors used to evaluate the interaction networks, differences in the abundance of plants, bee nesting sites and flight capacity could influence the results. Thus, for the first question, we used information from 11 canga patches, and for the second question, we used information from 9 canga patches.

**Question 1.** *Do the size and isolation of canga patches explain pollination syndrome frequency and plant composition*?

We built a dataset that contained information on the plants surveyed in the cangas of Carajás [39]. The canga patches were visited monthly by a large number of botanical researchers for two years, from March 2015 until December 2017. The main goal of this field work was to build the Flora of Carajás, for which 856 species of flowering plants were surveyed in the same canga patches [37]; using this information, we were able to define the pollination syndromes of 771 flowering plant species using the criteria proposed by Fenster et al 2004 and Rosas-Guerrero et al 2014. The floral traits used to define the pollination syndromes are listed in Table 2. For the determination of these attributes, we consulted specific literature [40–44], the internet, and botanical specialists on canga flora.

**Table 2:** Pollination syndromes and their characteristics (modified from Faegri & van der Pijl 1979 and Rosas-Guerrero et al. 2014) in relation to plant species on the ferruginous outcrops located in the tropical Amazon forest of Carajás.

Pollination syndrome	Aperture	Color	Odor strength/type	Shape	Orientation	Size/symmetry	Nectar guide/sexual organ	Reward
Anemophily/wind	diurnal; nocturnal	green whitish	imperceptible	brush	upright	amorphous	absent	absent
Cantharophily/beetles	diurnal; nocturnal	brown; green; white	strong/fruity; musky	dish	horizontal; upright	large/radial	absent/exposed	food tissue; heat; nectar; pollen
Entomophily/insects*	diurnal; nocturnal	bright colors						nectar; pollen
Phalaenophily/moths	nocturnal	white	moderate; strong/sweet	bell; brush; tube	horizontal; pendent/upright	medium; large; large/radial	absent/closed	nectar
Melittophily/bees	diurnal	blue; pink; purple; white; yellow	imperceptible; weak/fresh; sweet	bell; dish; tube; flag; gullet	horizontal; pendent; upright	small; medium; large/bilateral; radial	absent; present/closed; exposed	fragrance; nectar; oil; pollen; resin
Myophily/flies	diurnal	brown; green; white; yellow	imperceptible; weak/fruity; sour	bell; dish	horizontal; upright	small/radial	absent; present/exposed	nectar; pollen
Ornithophily/hummingbirds	diurnal	orange; pink; red; yellow	imperceptible	brush; tube; flag; gullet	horizontal; pendent; upright	medium; large/bilateral; radial	absent/exposed	nectar
Psychophily/butterflies and diurnal moths	diurnal	blue; orange; pink; red; yellow	weak/fresh	bell; brush; tube	horizontal; upright	small; medium; large/radial	absent; present/closed	nectar
Chiropterophily/bats	nocturnal	dark red; green; white	moderate; strong/fruity; musky; sour	bell; brush; dish; gullet	horizontal; pendent; upright; (far ground)	large; large/bilateral; radial	absent/exposed	food tissue; nectar; pollen

* The entomophily syndrome involves a set of traits that make flowers attractive to several insects, and it is therefore not possible to determine a particular insect group.

The dissimilarities in plant richness and pollination syndrome composition between each pair of canga patches were calculated with the Jaccard and Bray-Curtis indices, respectively [45]. We used the dissimilarities between each pair of canga patches to construct two dissimilarity matrices, one for each variable (plant richness and pollination syndrome composition). Then, we used the Mantel test to verify the correlation between the dissimilarity and geographic distance (interpatch distances) matrices. Results of Mantel tests are related to correlations between matrices and are represented as Pearson's R (or r). Moreover, we tested whether plant richness was affected by canga patch size by performing a linear regression between plant richness and canga size, which is represented as the coefficient of determination (R^2^), i.e., the proportion of the variance in the dependent variable that is predictable by the independent variable(s).

We also used generalized linear models (GLMs) to evaluate the effects of the size and isolation of the canga patches on the frequency of pollination syndromes. The frequency of a particular pollination syndrome is the number of plant species that are classified as exhibiting the specific syndrome. Because plant richness affects the frequency of pollination syndromes, we corrected for this plant richness bias by creating a binomial distribution for each pollination syndrome, in which success (1) was the number of plants in each canga patch classified as having a particular pollination syndrome (absolute frequency) and failure (0) corresponded to all the other plant species in the same canga patch that were not classified as having this particular pollination syndrome. In the GLM analyses, we built four models: 1) the null model, 2) the model with canga size, 3) the model with canga isolation, and 4) the additive model with both canga size and canga isolation.

We used the Akaike information criterion for small sample sizes (AICc) to select the most plausible model for each set, and models with lower delta AICc <2 were considered equally plausible [46, 47].

**Question 2.** *Do the size and isolation of canga patches explain interaction network structure and the diversity of interactions and pollinators*?

### Field work

The survey was carried out in the same cangas of Carajás. We selected canga areas where the bees that visited the open flowers were collected in a way that allowed us to determine which species of bees interacted with which plant species. These interactions are represented as interaction networks, where the partner species are connected by links. Within each canga patch, the sample area contained four 5 x 5 m plots and one 1 km x 3 m transect. These areas were visited monthly over the course of a year. Each month, we spent 9 days sampling the plant-pollinator interactions, and one canga patch was sampled per day. Each flowering plant (individual) was observed for 5 minutes on each sampling day. Each flower visitor that touched the reproductive parts of a flower was considered a pollinator and was collected. Each bee species was identified and deposited in the entomological collection of the Museu Paraense Emilio Goeldi.

### Data analysis

To answer Q2, we analyzed the relationships between the plant-pollinator interaction network indices and the size and isolation of the canga patches. Moreover, we wanted to understand whether species sharing in different canga patches was related to shared interactions; that is, if the same species are present in all canga patches, are the interactions also the same? We also wanted to determine whether species (bees and plants) and interactions with a higher degree (a larger number of links—see below) were present in a larger number of canga patches.

We constructed weighted (frequency of visits) interaction matrices with plants in the rows (P) and bees in the columns (B). For each bee-plant interaction, we recorded the number of individuals that visited the plant in the respective cell. Thus, the links are the number of individuals of each bee species that visited a specific plant species. We used four indices to describe network structure: 1) the measure of network specialization, the H_2_’ index [48], which varies from zero (complete generalization) to one (complete specialization) [49] and is useful for comparisons across different interaction webs and is not affected by network size or sampling effort [48]; 2) interaction diversity, which is based on the Shannon index; 3) pollinator richness; and 4) network nestedness (NODF metric), which varies from 0 (nonnested) to 100 (perfectly nested). The four indices were calculated using the bipartite package [49] in the R program [50]. We used GLM analysis to evaluate the relationship between the network indices and the landscape features. The landscape variables were log-transformed. For NODF and H_2_’, we used the beta distribution; for the diversity interaction, we used the gamma distribution; and for pollinator richness, we used the Poisson distribution [51]. In all GLM analyses, we used four models: 1) the null model, 2) a model with canga size, 3) a model with canga isolation and 4) an additive model with both canga size and canga isolation. These four models were compared, and we show only the best models in the results. To perform the GLM analyses, we used the bbmle package [52] in the R program [50].

We performed the Mantel test to verify whether interaction turnover was explained by species turnover. For this test, we constructed two distance matrices, one for bee species and another for plant species. Each matrix had 9 rows and 9 columns containing the information regarding canga patches. By definition, the distance of one canga from itself is 0 (main diagonal of the table). Thus, the upper diagonal contained information on the number of interactions shared by two canga patches, and the lower diagonal contained the number of species shared by these same two canga patches. Thus, paired matrices were built with pairwise information for each of the two canga patches for all canga patches. We performed 999 permutations for all Mantel tests, and we used the vegan package [53] in the R program [50].

We also verified whether the most generalist species and the most frequent interactions were ubiquitous. To determine which species were generalists or specialists, we measured the degree of the species, which is the number of interactions carried out by each species. We performed three linear regressions: 1) between the degree of each bee species and the number of canga patches from which each bee species was collected; 2) between the degree of each plant species and the number of canga patches within which each plant species was observed; and 3) between the interaction frequency (the number of members of bee species *I* that were observed visiting plant species *j*) and the number of canga patches within which each interaction was observed. To perform the linear regression, we confirmed that the variables were fitted to a normal distribution. We tested the normality and homogeneity of variances of the studied variables using the Shapiro-Wilk test and F-test in the R program [50].

## Results

**Question 1.** *Do the size and isolation of canga patches explain pollination syndrome frequency and plant composition*?

**Fig 2:**
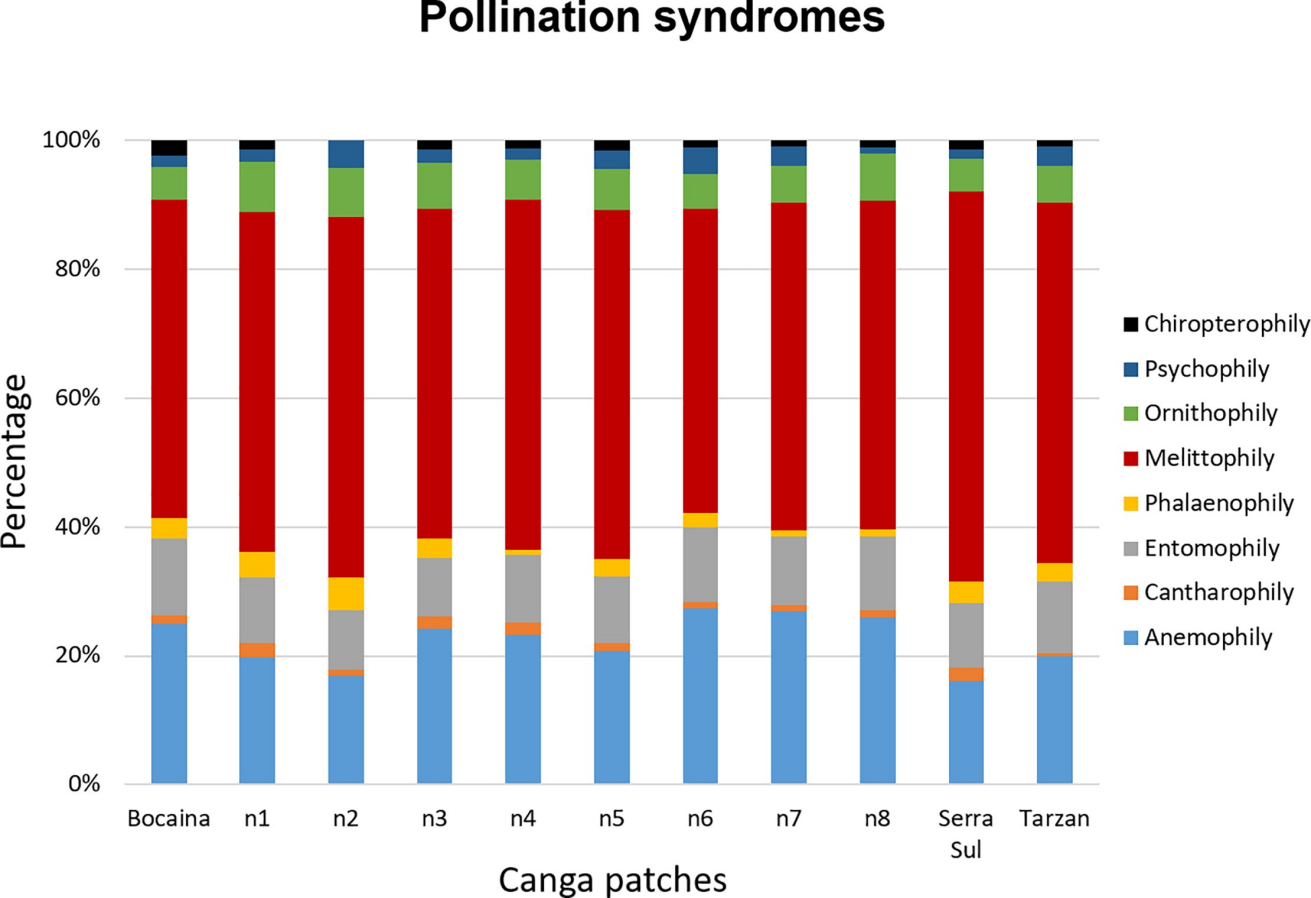
Percentage of pollination syndromes in all areas of ferruginous outcrops within the tropical Amazon forest of Carajás.

We defined eight pollination syndromes among the 771 plant species occurring in the eleven studied canga patches. Serra Sul and Bocaina were the largest canga patches but also the most isolated ones (Table 1). The richest canga patch in terms of plant species was Serra Sul, with 612 species, followed by N1, with 360 species (Table 2). The most frequent pollination syndrome was melittophily (50%) (Fig 2). All pollination syndromes were recorded in the eleven canga patches, except that chiropterophily was not recorded in N2.

Plant richness increased with patch size (F = 43.91; d.f. = 9; p = 0.0009 R^2^ = 0.81). However, the dissimilarities in plant species and pollination syndromes were not correlated with canga isolation (Mantel test p = 0.21; r = 0.17 and p = 0.2; r = 0.18, respectively).

When we analyzed the frequency of pollination syndromes, the null model provided the best fit for all pollination syndromes (see SS 1 in S1 File).

**Question 2.** *Do the size and isolation of the canga patches explain interaction network structure and the diversity of interactions and pollinators*?

**Fig 3:**
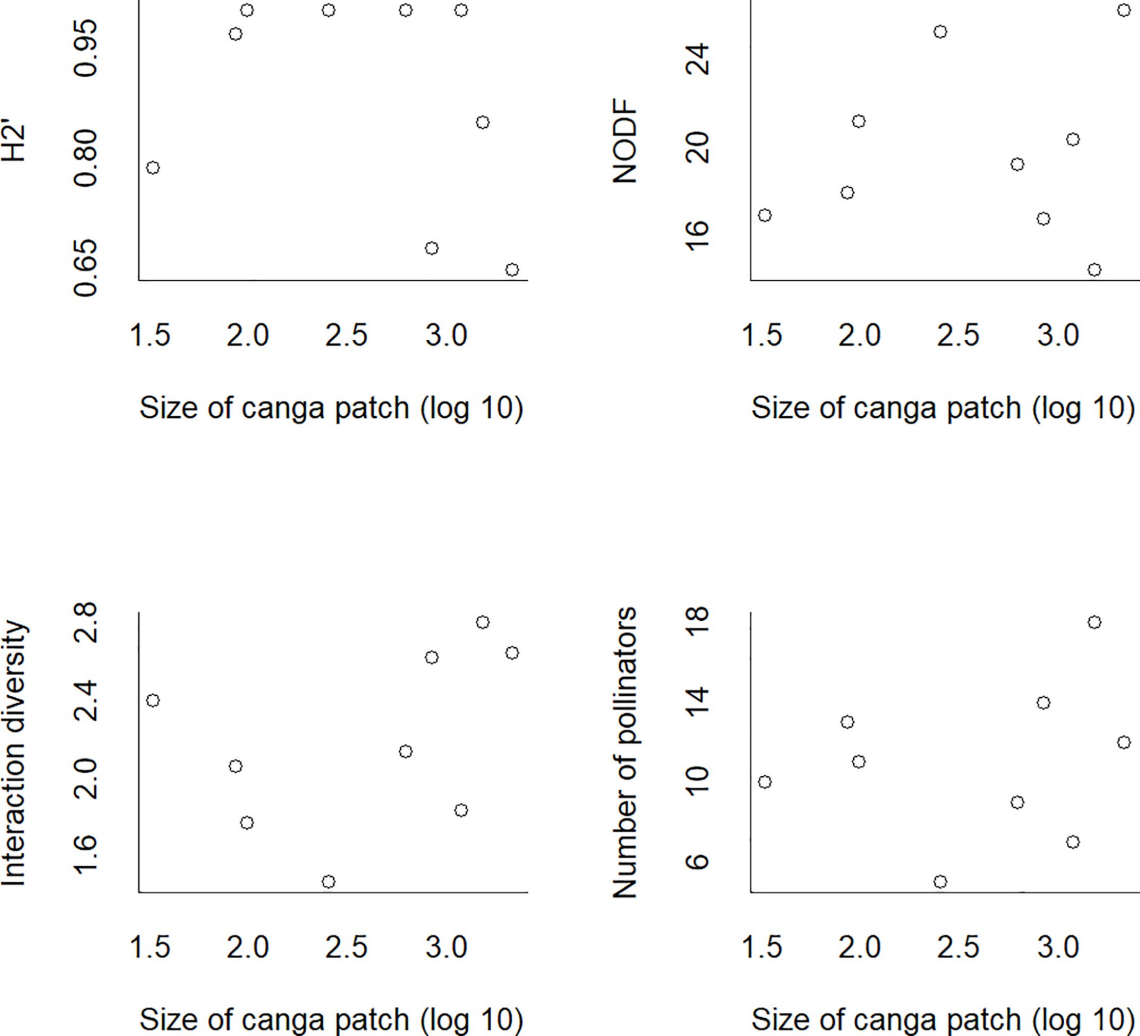
The structure of plant-pollinator networks in Carajás was not affected by the size of the canga patches. The specialization index (H_2_’), nestedness (NODF), interaction diversity and pollinator richness were stable among habitat sizes.

**Fig 4:**
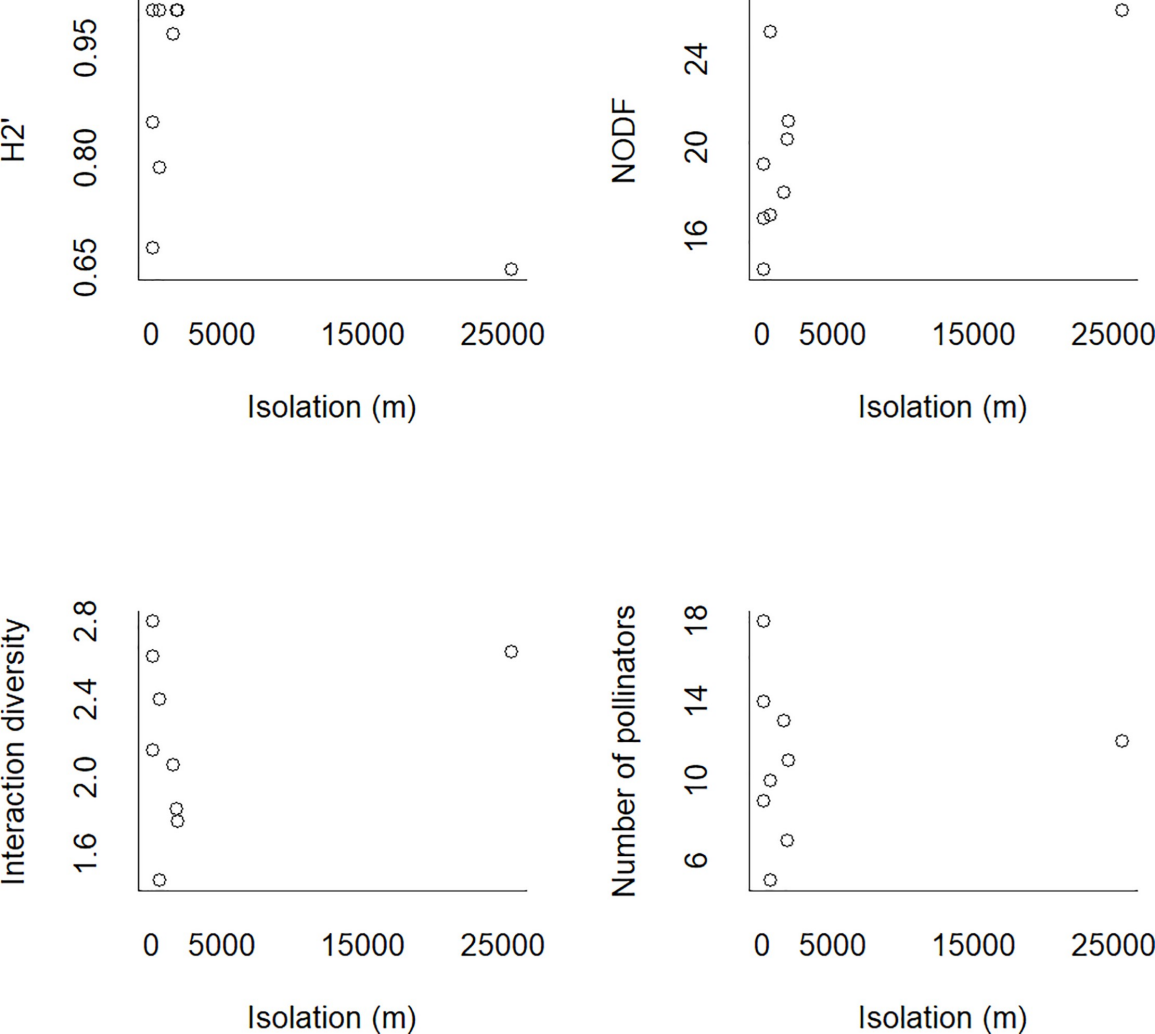
The structure of the plant-pollinator networks in Carajás was not affected by the isolation of the canga patches. The specialization index (H_2_’), nestedness (NODF), interaction diversity and pollinator richness were stable despite isolation.

**Fig 5:**
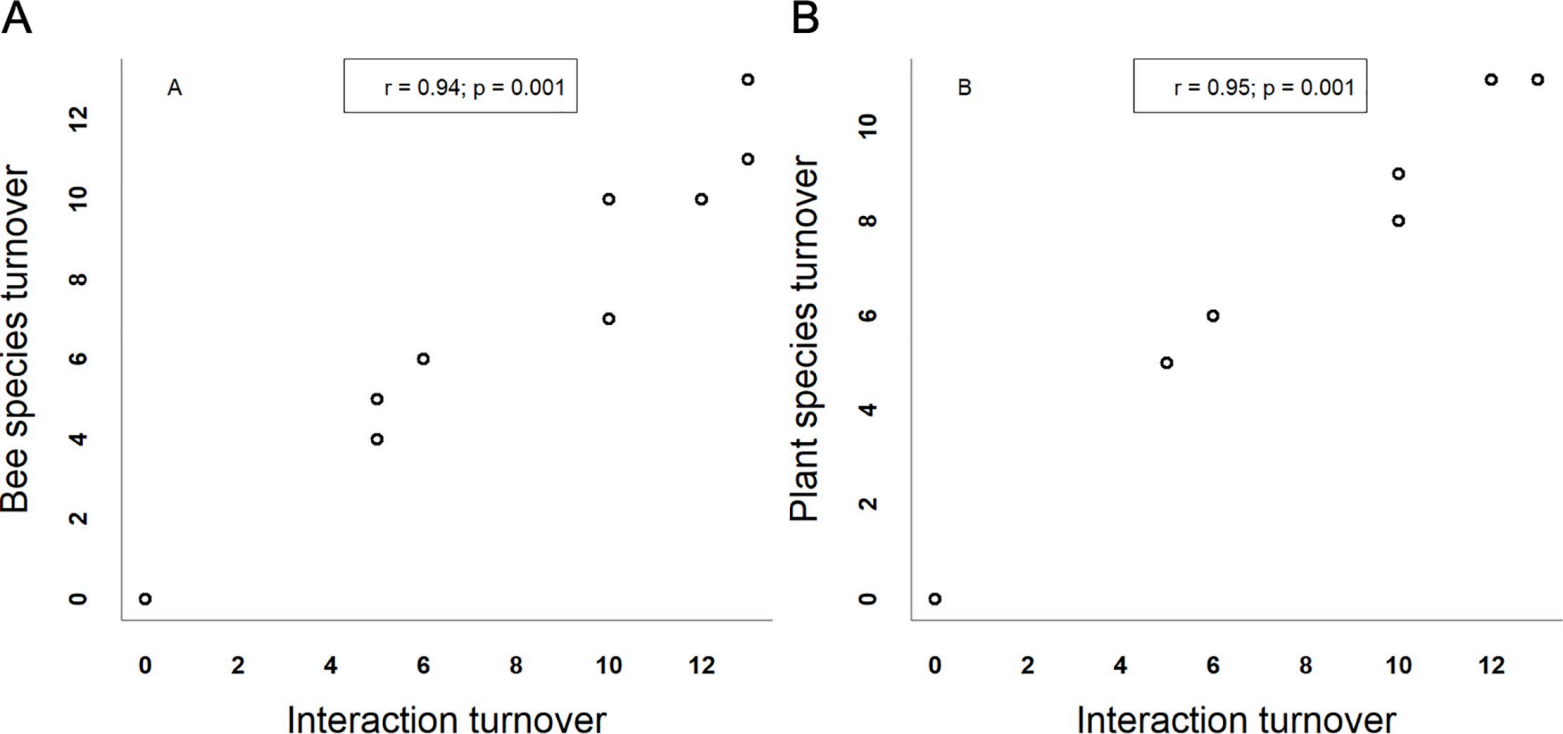
The turnover of interactions across the canga patches is explained by species turnover. A) The canga patches that shared bee species shared species that interacted, and B) the same pattern was found for plant species.

**Fig 6:**
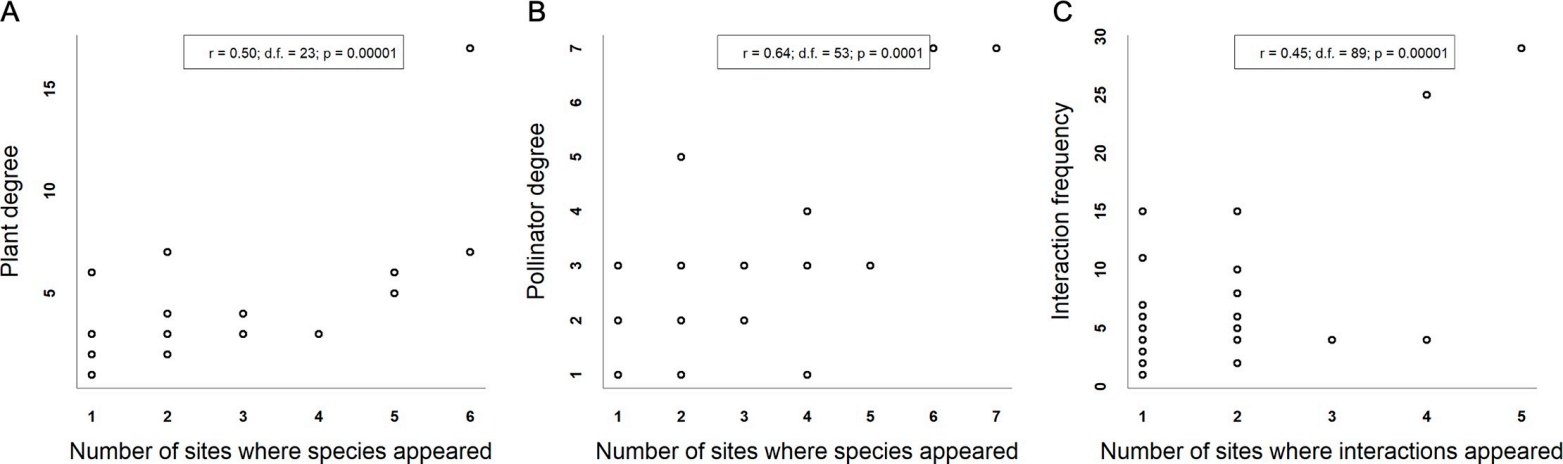
A linear regression between species degree/interaction frequency and the number of canga patches for each species was fit. The species degree is the number of interactions carried out by each species. Linear regression A) between the degree of each plant species and the number of canga patches within which each plant species was observed; B) between the degree of each bee species and the number of canga patches from which each bee species was collected; and C) between the interaction frequency (the number of members of bee species *I* that were observed visiting plant species *j*) and the number of canga patches within which each interaction was observed.

Individuals of 53 bee species were observed visiting flowers of 27 plant species in the nine canga patches and collected. Four plant species were visited by individuals of only one bee species. Nineteen bee species interacted with only one plant species. The N7 network was the smallest, with only four bee and five plant species. On the other hand, the S11A network was the largest, with 18 bee and 8 plant species. In general, the networks were specialized (H_2_’ = mean ± SD: 0.88 ± 0.14). The Bocaina network showed lower specialization (H_2_’ = 0.67), and the N1, N6, N7 and S11C networks showed higher specialization (H_2_’ = 1). The networks exhibited a nonsignificantly nested network topology (all p-values > 0.05); the NODF values were low and ranged from 14.5 to 26.21. The S11A network had the greatest interaction diversity (ID = 2.8), and the N7 network had the lowest interaction diversity (ID = 1.4). The S11A and N7 networks were also the richest and the poorest, respectively, in terms of the number of bee species. Canga patch size (Fig 3) and isolation (Fig 4) were not related to any of the indices describing network structure. For the four network indices, the null model provided the best fit (see SS 2 in S1 File). There was high interaction turnover across the canga patches. This interaction turnover was related to plant turnover (Mantel test p = 0.001; r = 0.95) and bee turnover (Mantel test p = 0.001; r = 0.94) (Fig 5). The ubiquity of the species was related to degree, meaning that the most generalist plant and bee species were found in all canga patches (F = 11.48; d.f. = 25; p = 0.002; r = 0.28 and F = 36.48; d.f. = 51; p = 0.00001; r = 0.4, respectively) (Fig 6A and 6B). The same result was found for interactions since the most frequent interactions were ubiquitous (F = 55.45; d.f. = 97; p = 0.00003; r = 0.35) (Fig 6C).

## Discussion

Our results indicate that the size and isolation of patches do not affect the plant-pollinator interactions in naturally isolated canga outcrops, since the frequency of pollination syndromes and the structure of the plant-pollinator networks were not affected by these two variables. Moreover, a core set of generalist species remained stable among the canga patches.

Contrary to our expectations, the results suggest that for naturally isolated canga patches, size and isolation do not affect interactions. The network structure found in the cangas of Carajás was spatially stable, and neither habitat size nor isolation affected nestedness, specialization, interaction diversity or pollinator richness. Additionally, we detected turnover of plant and bee species among the canga patches, and species turnover was a main driver of interaction turnover. Despite these changes in species and interactions, network structure was not affected.

Our results suggest that the Amazon forest is not a barrier to the species found in the study area. In Carajás, most of the forest that separates the canga patches is well preserved, and in this case, in comparison to the agricultural or urban areas that are usually found between the fragmented areas, this type of matrix is likely to be less impeding to species movement [54]. Moreover, approximately 60% of the 856 flowering plant species in Carajás can also be found in the surrounding forest [37], supporting that long distances between canga patches do not truly imply isolation. In fact, a recent study on species of *Ipomoea* (Convolvulaceae) that occur in cangas showed that the Amazon forest did not prevent the gene flow of this plant species between canga patches and that genetic diversity was not affected by the size of the cangas [55]. Other previous studies have shown that the matrix can also influence resource availability [56], animal dispersion [57], habitat (fragment) occupation [58, 59], and the distribution and population dynamics within a fragment [60, 61]. The similarity between the matrix and fragments is also an important characteristic that facilitates gene flow and the dispersion of animals [60]. Thus, the Amazon forest may play an important role in maintaining functional diversity among canga patches, as matrix permeability is an important factor that prevents fragment size and isolation from affecting interactions.

Our results regarding pollination syndromes revealed certain similarities with those reported in previous studies. First, melittophily has also been shown to be an important syndrome in Brazilian dry forest, since 43% of the plant species were classified as being pollinated by bees [62]. Second, in small Atlantic forest fragments, the frequencies of species and individuals showing melittophily, entomophily and anemophily syndromes were high [21]. These three syndromes (melittophily, entomophily and anemophily) are considered to involve pollination by generalist vectors, including small bees, moths, butterflies, various small insects, and wind [63]. We found that these syndromes were also ubiquitous in the cangas of Carajás, with 88% of the plant species recorded as having them. Considering other studies on interaction networks, both generalist plant and pollinator species have been documented as being less susceptible to variation in area size and isolation than specialists and responsible for maintaining the structure of mutualisms over space and time [10, 64–66]. Generalist pollinator species maintain plant communities, as reported in other plant-pollinator studies [10, 67, 68]. Generalist bee species are also important for maintaining the robustness of the structure of pollination networks and are responsible for sustaining plant specialist species [69, 70]. In general, in interaction networks, generalist plant and pollinator species are more abundant than specialist species [69, 70]. Generalist plant and pollinator species may also play a central role in driving evolution and coevolution in species-rich assemblages, pushing them toward high complementarity and, above all, convergence [71]. Thus, generalist plant and pollinator species are ubiquitous and capable of maintaining interactions despite changes in their habitats; in addition, they are the main species with which plant and pollinator specialists interact, preserving the structure of interaction networks over space and time.

Despite the large number of plant species described in the cangas of Carajás [37], only a few species were observed interacting with bees. Studies of interaction networks, especially those involving plant-pollinator interactions, require extensive field work throughout the flowering season of plants to avoid mismatches, such as forbidden links [72]. However, the current study is the first work on plant-pollinator interactions in this region and can pave the way forward for future studies aimed at increasing the knowledge on this region. Moreover, pollination syndrome analysis is considered controversial because several pollinator species can interact with some plant species [27]. The recognition of specific pollinators ideally requires the observational identification of visitors to flowers and an additional assessment of the efficiency of pollen transfer and subsequent fertilization [73]. However, pollination syndromes have proven to be an important proxy for understanding interaction networks [68]. This approach can provide preliminary insights into mutualistic interactions, especially in areas with high diversity and important gaps in knowledge, such as the tropical Amazon biome.

## Conclusion

Our study shows that plant-pollinator interactions in naturally isolated outcrops are more stable than those in human-induced habitat fragments. Furthermore, despite the turnover in species among the canga patches, the generalist species tend to be ubiquitous and are key to maintaining the stability of plant-pollinator interactions. We argue that the matrix surrounding the canga patches is not a barrier and represents a key factor driving the interactions between the plants and pollinators in this area.

## Supporting information

S1 File (DOCX)
