# Supplementary material for: Size and isolation of naturally isolated habitats do not affect plant-bee interactions: A case study of ferruginous outcrops within the eastern Amazon forest
Source: PLoS One. 2020 Sep 11;15(9):e0238685. doi: 10.1371/journal.pone.0238685 (PMC7485833; doi:10.1371/journal.pone.0238685)
Supplement: S1 File — (DOCX) [file pone.0238685.s001.docx]

| **Pollination syndrome** | **Model** | **dAICc** | **df** | **weight** |
| --- | --- | --- | --- | --- |
| Anemophily | Null model | 0 | 1 | 0.61 |
| Cantharophily | Null model | 0 | 1 | 0.51 |
| Entomophily | Null model | 0 | 1 | 0.62 |
| Melittophily | Null model | 0 | 1 | 0.68 |
| Ornithophily | Null model | 0 | 1 | 0.66 |
| Psychophily | Null model | 0 | 1 | 0.62 |
| Phalaenophily | Null model | 0 | 1 | 0.59 |
| Chiropterophily | Null model | 0 | 1 | 0.55 |

SS 1. The null model was the best fitted model for the all pollination syndromes studied according the dAICc.

| **Model** | **dAICc** | **df** | **weight** |
| --- | --- | --- | --- |
| H_2_’ null model | 0 | 2 | 0.79 |
| H_2_’ size model | 3.8 | 3 | 0.11 |
| H_2_’ isolation model | 4.7 | 3 | 0.07 |
| H_2_’ size and isolation model | 8.4 | 4 | 0.01 |
| NODF null model | 0 | 2 | 0.51 |
| NODF size model | 4.7 | 3 | 0.05 |
| NODF isolation model | 0.4 | 3 | 0.42 |
| NODF size and isolation model | 7.3 | 4 | 0.01 |
| Interaction diversity null model | 0 | 2 | 0.75 |
| Interaction diversity size model | 3.4 | 3 | 0.14 |
| Interaction diversity isolation model | 4 | 3 | 0.10 |
| Interaction diversity size and isolation model | 10.3 | 4 | 0.004 |
| Number of pollinators null model | 0 | 1 | 0.69 |
| Number of pollinators size model | 2.9 | 2 | 0.16 |
| Number of pollinators isolation model | 3.4 | 2 | 0.12 |
| Number of pollinators size and isolation model | 7.7 | 3 | 0.01 |

SS 2. GLM models results of interaction network metrics and size and isolation of canga patches.
